# Benchmarking urine storage and collection conditions for evaluating the female urinary microbiome

**DOI:** 10.1038/s41598-019-49823-5

**Published:** 2019-09-16

**Authors:** Carrie E. Jung, Jessica Chopyk, Ji Hyun Shin, Emily S. Lukacz, Linda Brubaker, Leila K. Schwanemann, Rob Knight, Alan J. Wolfe, David T. Pride

**Affiliations:** 1 Department of Obstetrics, Gynecology & Reproductive Sciences, University of California, San Diego, 92093 USA; 2 Department of Pathology, University of California, San Diego, CA 92093 USA; 3 Departments of Pediatrics, Bioengineering, and Computer Science and Engineering, University of California, San Diego, 92093 USA; 4 Department of Microbiology and Immunology, Loyola University Chicago, Maywood, IL 601534 USA; 5 Department of Medicine, University of California, San Diego, CA 92093 USA

**Keywords:** Clinical microbiology, Microbial ecology, Microbiome

## Abstract

Standardized conditions for collection, preservation and storage of urine for microbiome research have not been established. We aimed to identify the effects of the use of preservative AssayAssure® (AA), and the effects of storage time and temperatures on reproducibility of urine microbiome results. We sequenced the V3–4 segment of the 16S rRNA gene to characterize the bacterial community in the urine of a cohort of women. Each woman provided a single voided urine sample, which was divided into aliquots and stored with and without AA, at three different temperatures (room temperature [RT], 4 °C, or −20 °C), and for various time periods up to 4 days. There were significant microbiome differences in urine specimens stored with and without AA at all temperatures, but the most significant differences were observed in alpha diversity (estimated number of taxa) at RT. Specimens preserved at 4 °C and −20 °C for up to 4 days with or without AA had no significant alpha diversity differences. However, significant alpha diversity differences were observed in samples stored without AA at RT. Generally, there was greater microbiome preservation with AA than without AA at all time points and temperatures, although not all results were statistically significant. Addition of AA preservative, shorter storage times, and colder temperatures are most favorable for urinary microbiome reproducibility.

## Introduction

The recent discovery of a unique bladder microbiome has contradicted the prevailing dogma that healthy urine is sterile^1–3^, and shifted the field to the paradigm that the urinary microbiome may play a role in bladder health and disease. Systematic DNA sequence-based investigation of the human microbiome began with the Human Microbiome Project^4^, a study that catalogued the microbiome inhabiting up to 18 different body niches of 300 people, but did not include the urinary bladder. Using a culture-independent technique, sequencing of the 16S rRNA gene^5^, the urinary microbiome was documented to differ in certain bladder conditions compared to controls^6–8^. The existence of a urinary microbiome enables researchers to seek a greater understanding of the microbes that inhabit the bladder, including improving understanding of when a well-known uropathogen such as *E*. *coli* will cause a conventional urinary tract infection (UTI), but also when an overgrowth of a urogenital pathogen or even a commensal organism in the bladder may be associated with disease^9^.

Analysis of the human microbiome is complex, with many layers that could decrease reproducibility due to variation between collection and storage methods across research laboratories and institutions. There have been several studies examining storage conditions of fecal specimens with preservatives having varying effects depending on which one was used^10–18^. The consensus of these studies is that storing at colder temperatures for shorter time periods is preferred. One study of vaginal samples showed that there were no significant differences in results in those processed immediately compared to those that were initially stored at −20 °C or −80 °C for a few weeks^19^; however, this study did not examine the effects of non-freezing storage temperatures on the specimens. It is critical to include storage conditions that do not include freezing in research studies because those conditions usually apply to clinic setting and community settings where specimens may be collected. Although many studies have characterized the urinary microbiome^20,21^, there is little guidance for researchers regarding collection and storage of urinary samples for microbiome analyses. Protocols vary in storage times, temperatures, and use of DNA preservative (such as the nucleic acid preservative AssayAssure®). These protocols may include varying storage temperatures from 4 °C to −20 °C at time periods from <4 hours to >24 hours to unspecified time periods prior to storage at −80 °C^1,2,22–26^. The impact of these variations on urinary microbiome readouts is not known. Because urine specimens are relatively low biomass, storage practices could have a larger impact on the urinary microbiome. Therefore, it is critical to assess whether there is variability introduced by methodology, define optimal techniques, and establish a standard protocol for collection and storage of these urinary samples.

With this study, we sought to establish a consistent starting point for urinary microbiome analysis by identifying storage and preservation conditions that optimize reproducible urinary microbiome analyses. We tested the effects of three variables on urinary microbiome specimens prior to ultimate storage of all the specimens at −80 °C until analysis, including multiple permutations of time (ranging from freezing immediately to 4 days later), temperature (ranging from room temperature to −20 °C), and the presence of a nucleic acid preservative (AssayAssure®).

## Results

### Sampling scheme to assess storage conditions

**Figure 1:**
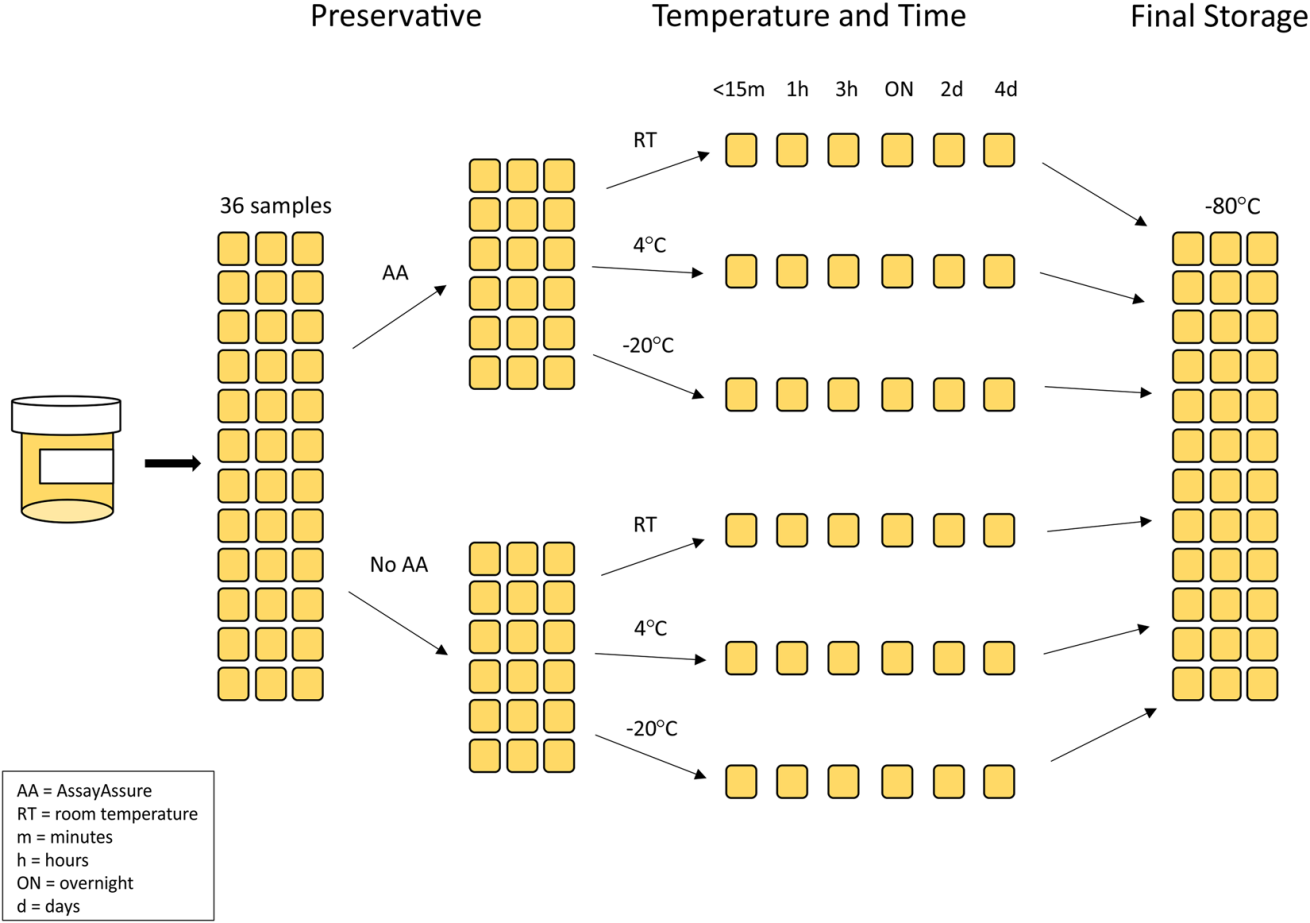
Storage schema flow diagram.

We recruited 10 female participants via word-of-mouth, mean ages 40.4 ± 3.9 years old (Table S1); each provided a midstream voided urine specimen. The specimens from each participant were immediately processed into 36 different 200 ul aliquots (Fig. 1). Half of the aliquots were immediately placed into 20 µl of AssayAssure (AA) preservative, and the other half were stored without AA. Therefore, for all storage conditions, we created an aliquot preserved with and without AA. We stored the aliquots at 3 separate temperatures, including room temperature (RT), 4 °C, and −20 °C. Aliquots also were stored for different time periods, including immediate (<15 minutes), 1 hour, 3 hours, overnight (approximately 16 hours), 2 days, and 4 days. At the end of each of these time periods, the aliquots were all stored at −80 °C for up to 30 days until they could be processed further to characterize their urinary microbiome.

### Effect of AA preservative

**Figure 2:**
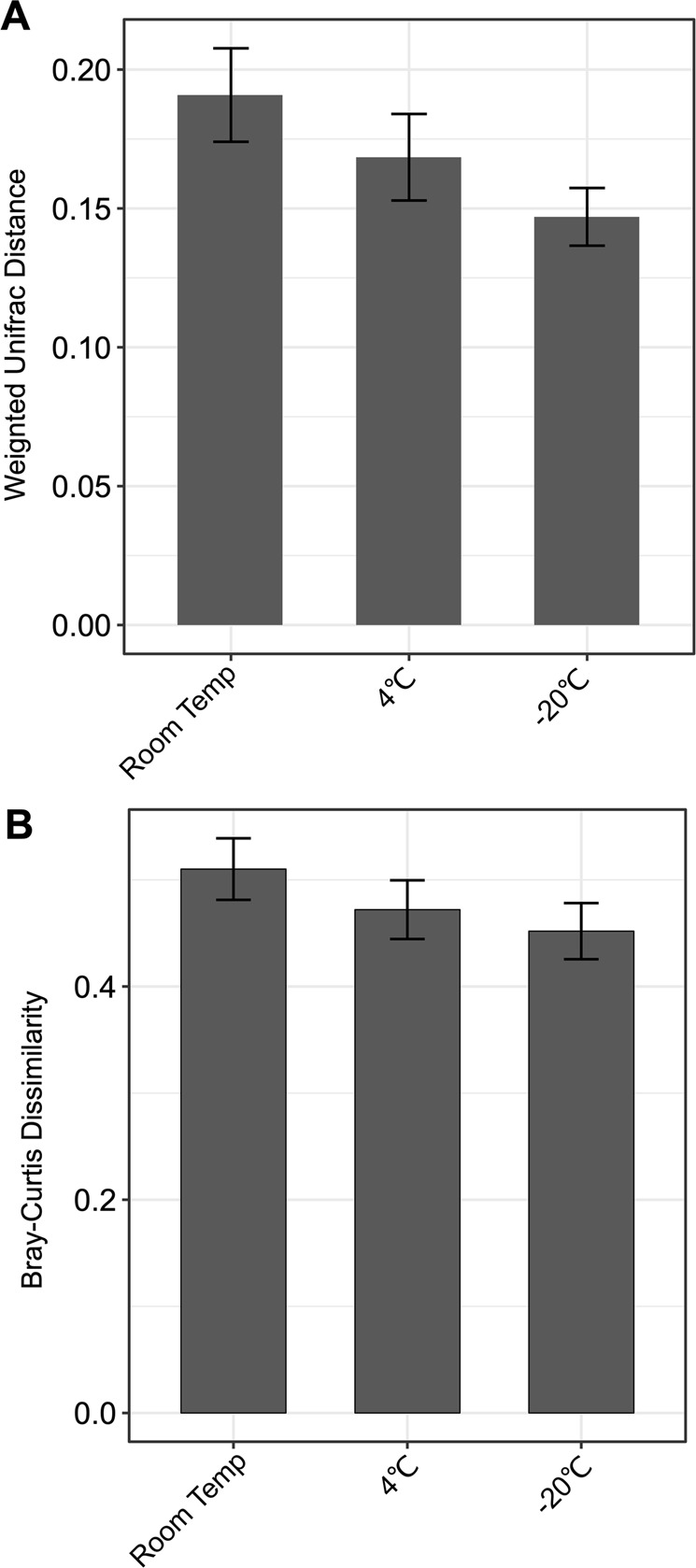
Bar graphs representing (**A**) weighted UniFrac distances (±standard error) and (**B**) Bray Curtis dissimilarity (±standard error) for urine specimens stored in AA preservative compared to those stored without preservative. Specimens stored at RT, 4 °C, or −20 °C are demonstrated on the x-axis, and weighted UniFrac distances or Bray Curtis dissimilarity are shown on the y-axis.

We characterized the bacterial community in each aliquot from all 10 participants by sequencing the V3–V4 segment of the 16S rRNA gene^27^. To determine the effects of AA in preserving the urinary microbiome, we calculated weighted UniFrac distances and Bray Curtis dissimilarity between matched specimens with and without AA. The samples were then grouped according to the temperature in which they were immediately stored to separate the effects of temperature on urinary microbiome contents (Fig. 2). The weighted UniFrac distances and Bray Curtis dissimilarity were generally higher in samples stored at RT. However these results were not significant and could reflect variability at each time point.

**Figure 3:**
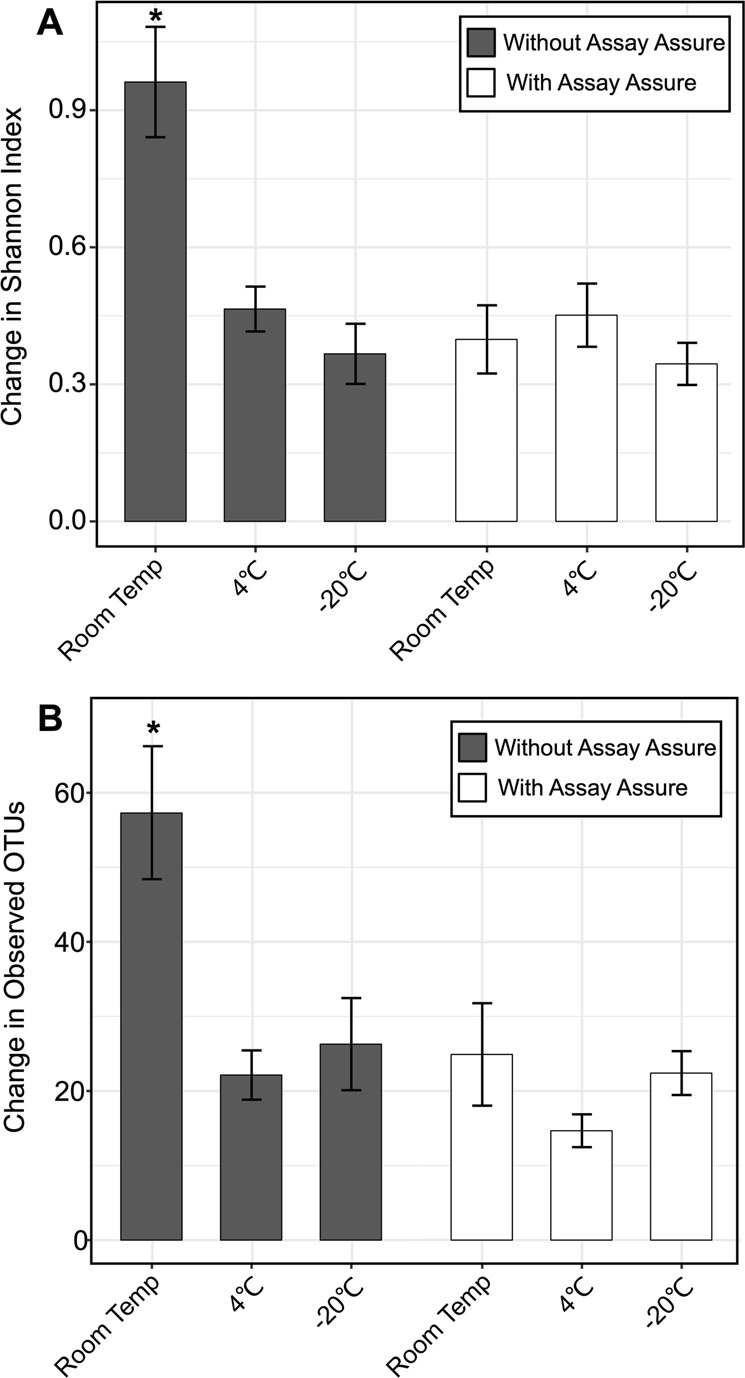
Bar graphs representing mean changes in the (**A**) Shannon Index (±standard error) and (**B**) Observed OTU values (±standard error) compared to the index time point (time point where urine specimens were collected and then immediately frozen at −80 °C) for urine specimens from all participants. Urine specimens preserved in AA are demonstrated by the white bars, and specimens without preservative are shown by gray bars. The mean change in alpha diversity measures are shown on the y-axis and specimen storage conditions are demonstrated on the x-axis. The ‘*’ represents values that are statistically significant with p-values ≤ 0.05 determined by an ANOVA with post-hoc Tukey HSD.

Prior studies have demonstrated differences in alpha diversity in the urinary tract in a number of conditions such as prostatitis, urinary incontinence, and kidney disease, with disease conditions often having lower diversity compared to normal controls^28–30^. To decipher factors that contribute to the differences observed in the urinary microbiome with and without AA, we calculated the number of distinct Operational Taxonomic Unites (Observed OTUs), as well as the Shannon index, a diversity metric that accounts for both abundance and evenness of the taxa present in a sample^31,32^. We determined the change in these alpha diversity metrics by comparing the index time point (immediately frozen at −80 °C) with each of the other time points, and plotted the change between the index and each individual time point (Fig. 3). We found that when specimens were stored in AA, the change in both alpha diversity metrics was similar regardless of whether the specimens were originally stored at RT, 4 °C, or −20 °C. However, when no AA was added, the change in both Shannon Index and the number of observed OTUs was significantly increased when the specimens were stored at RT compared to those stored at 4 °C and −20 °C.

We also examined changes in the bacterial community composition from the 10 participants with and without AA at the 3 different temperatures (RT, 4 °C, −20 °C). We used differential abundance testing using balances in gneiss^33^ to identify microbes with different relative abundances at the same temperature with and without AA. We identified different sets of microbial OTUs with significant differences in their relative abundances at each temperature (Table 1). At RT with AA, we found that there were significant decreases in the proportions of *Enterococcus*, *Klebsiella*, *Pseudomonas*, and *Akkermansia* species. At 4 °C with AA, we found significant differences in the proportions of *Lactobacillus*, *Gardnerella*, *Ureaplasma*, *Stenotrophomonas*, and *Delftia* species. Specifically, *Delfti*a, *Stenotrophomonas*, and *Ureaplasma* species significantly increased in representation when specimens were preserved with AA. When the urine specimens were stored at −20 °C with AA there were also decreases in *Anaerobacillus*, *Klebsiella*, *Paracoccus*, *Citrobacter*, *Bacillaceae*, and *Anerococcus* species. We also tested the AA alone to ensure that it was not contaminated, and found no evidence of *Delftia* species or other bacteria in the AA (data not shown).

**Table 1:** Differentially abundant taxa.

Room Temp^a^	4 °C^a,b^	−20 °C^a,b^
↑Lactobacillus	↑↓Lactobacillus	↑↓Lactobacillus
↓Klebsiella		↓Klebsiella
↓Enterococcus		
↓Pseudomonas		
↓Akkermansia		
	↑Ureaplasma	
	↑↓Gardnerella	
	↑Stenotrophomonas	
	↑Delftia	
		↓Anaerobacillus
		↓Paracoccus
		↓Citrobacter
		↓Bacillaceae
		↓Anaerococcus

^a^Up arrows indicate OTUs with greater abundance with AA.

^b^Up and down arrows together indicate taxa with representative OTUs that increased and representative OTUs that decreased in abundance with AA.

### Effects of storage time and temperature on the urinary microbiome

**Figure 4:**
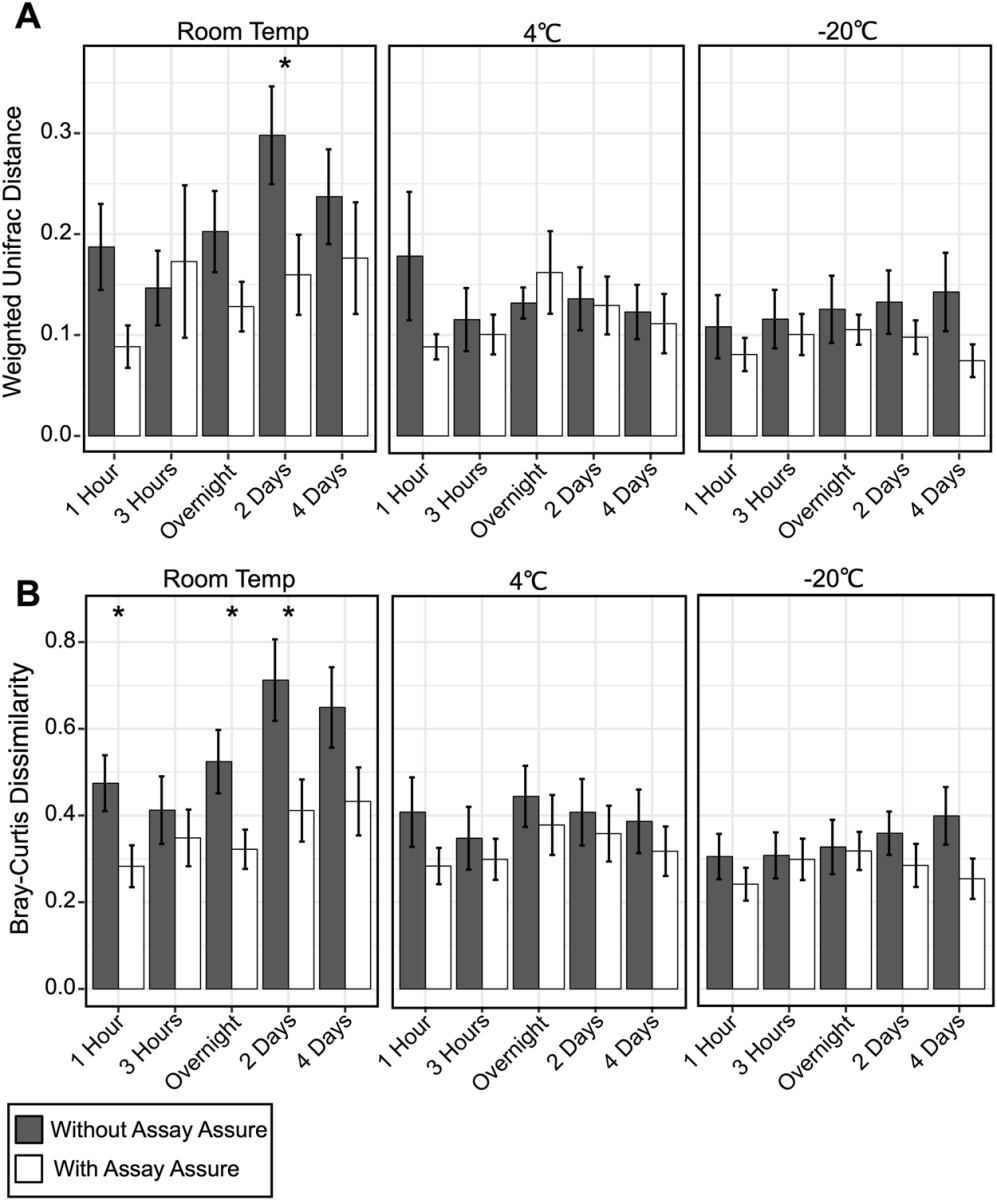
Bar graph representing (**A**) weighted UniFrac distances (±standard error) and (**B**) Bray Curtis dissimilarity (±standard error) for specimens stored at RT, 4 °C, and −20 °C with and without AA. Urine specimens preserved in AA are demonstrated by the white bars and specimens without preservative are shown by gray bars. The y-axis represents the weighted UniFrac distances and Bray Curtis dissimilarity, and the x-axis represents the time specimens were stored prior to transfer to −80 °C. The ‘*’ represents statistically significant values (p-values ≤ 0.05) using a two-tailed t-test.

Our data demonstrates that temperature played a significant role in the change in diversity of the urinary microbiome with and without AA (Fig. 3). Therefore, we evaluated the effects of storage time at each of the different temperatures on the urine microbiome. We calculated weighted UniFrac distances and Bray Curtis dissimilarity between each time point (1 hour, 3 hours, overnight, 2 days, and 4 days) and the corresponding index time point (immediately frozen at −80 °C) and compared the samples pairwise and across time (Fig. 4). For samples both with and without AA stored at −20 °C and 4 °C there were no significant changes in weighted UniFrac distances and Bray Curtis dissimilarity across time (Panels A and B). However, for those stored at RT without AA we did note a general increase in weighted UniFrac distances and Bray Curtis dissimilarity through time, although this was not significant at all time points. Additionally, when comparing the samples stored with and without AA, the Bray Curtis dissimilarity was significantly higher at 1 hour, overnight, and 2 days (Panel B). Similarly, for the weighted Unifrac distances the samples stored without AA were significantly higher at 2 days (Panel A). These results indicate that in the absence of AA, significant changes occurred in the urinary microbiome at RT, but much of the microbiome was preserved at 4 °C and −20 °C.

We also characterized the change in alpha diversity across time in the urine specimens processed with and without AA (Fig. S1). For the urine specimens preserved in AA, we generally observed less change in alpha diversity through time than observed for the specimens without AA, especially at RT (with the exception of the 3 hr time point with AA, which contained a single outlier). Moreover, when comparing Shannon diversity values and Observed OTUs to the index time point there were significant differences (Fig. S2, Panels A and B). Specifically, we observed a significant decrease in the Shannon Index at 2 days and 4 days in the samples stored at RT without AA compared to the index time point.

Finally, we compared the relative abundance of the most dominant bacterial phyla *Firmicutes*, *Actinobacteria*, *Proteobacteria*, and *Bacteriodetes* across time in the urine specimens processed with and without AA (Fig. S3). When compared to the index time point the only significant change in relative abundances was a decrease in the relative abundance of *Bacteriodetes* at RT for 2 days without AA.

### Microbiome variation in participants with and without AA

**Figure 5:**
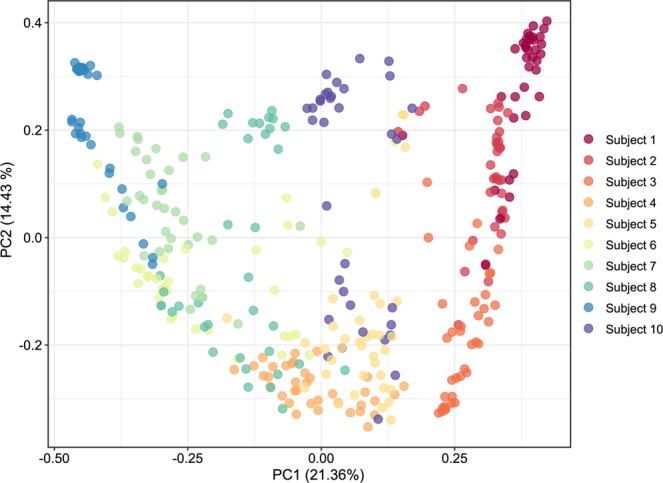
Principal coordinates analysis based on weighted UniFrac distances of urine specimens from all 10 participants studied.

We determined whether there may be individual-specific features of the urinary microbiome in the participants in this cohort by assessing beta diversity amongst the microbiomes of each participant and time point and visualized the output using Principal Coordinates Analysis (Figs 5 and S4). Many of the participants’ urine specimens clustered together and somewhat distinctly from the other participants, suggesting that there were features unique to each individual. Moreover, when separated by participants (Fig. S4) we found that individuals responded differently to the addition of AA. In the case of participants #1, #3, #6, #8, #9 samples with AA clustered separately from those without AA.

We next used differential abundance testing^33^ to identify which times and temperature conditions had the greatest variability. For most participants, the greatest variability in composition occurred for specimens stored at RT, as is seen for participants #1, #2, #3, #5, #6, and #8 (Fig. S5). These results support the contention that individual-specific urinary microbiome features and taxonomy may be least well preserved at RT.

Because of the individual-specific nature of the urine microbiome in each participant, we also examined differences in the proportion of microbes in each participant using differential abundance testing using balances in gneiss (Table S2). In participant #1 *Escherichia*, *Bacteroides*, and *Faecalibacterium* had greater abundance without AA. In participant #2, *Lactococcus* had greater abundance without AA. In participant #3, *Gardnerella* and *Streptococcaceae* had different abundances with and without AA. In participants #1, #2, #5, #6, and #7, *Stenotrophomonas* had greater abundance with AA. These data support that there were microbiome differences unique to many of the participants when urine specimens were preserved with and without AA. There were a small number of taxa that had greater abundance with AA in some individuals, but had diminished abundance with AA in others (Table S2).

## Discussion

Comprehensive studies to benchmark criteria for preservation and storage of urine specimens have been lacking. Because the field of urine microbiome research is still relatively new, it is prudent to establish a set of preference practices for urine specimens as the field moves forward. This study attempts to establish optimal storage and preservation techniques including the utility of AA as a preservative of the urinary microbiome and feasibility of storage at various times and temperatures researchers may employ before optimally storing at −80 °C. This is especially important, as the urinary microbiome may be particularly susceptible to variation due to the low biomass available for preservation.

Complicating any analysis of the urinary microbiome is the degree to which microbes may vary by individual. We observed differences in urinary microbiomes in each of the participants studied (Figs 5, S4, S5 and Table S2). These individual-specific characteristics create greater uncertainty in determining how preservatives, time and temperature affect the urinary microbiome. For example, when examining the cohort in aggregate we identified significant differences in the relative abundances of several taxa, including species of *Lactobacillus*, *Klebsiella*, *Delftia*, *Gardnerella*, and *Enterococcus*. However, when evaluating individuals separately, we identified additional differences in *Escherichia*, *Lactococcus*, and *Streptococcus* species, among others. These differences were attributable to the use of AA; however, whether a bacterium was more or less abundant in each individual in the presence of AA was not always predictable.

There were substantial differences in the urinary microbiomes when preserved with or without AA; however, those results were significantly influenced by time and temperature. This was most evident at RT with and without AA, where the only statistically significant differences in this study were observed for specimens stored without AA, with the effect being more pronounced at later time points. For instance, we identified greater abundances of potential uropathogens *Klebsiella* and *Enterococcus* species in urine at RT without AA, but not at RT with AA. These findings suggest that the addition of AA reduces overgrowth of these uropathogens when urine is stored at RT. Cultivation of urine stored at RT is the standard of care at many institutions^34^, which could result in overrepresentation of uropathogens. While there were significant microbiome differences at RT without AA, we did not observe many of these differences when the urine was stored for the same time periods at 4 °C and −20 °C. These data suggest that much of the benefit of AA as a preservative likely occurred at RT. We did not characterize length of storage at −80 °C, so we cannot rule out that storage times at −80 °C could have impacted results. Our data indicate that AA serves a preservative role in urinary microbiome analysis with specimens with the shortest storage times and stored at colder temperatures most reflective of baseline microbiomes.

Changes in alpha diversity appeared to be significantly increased during storage at RT without AA (Fig. 3). In contrast, we observed no significant changes in alpha diversity in the specimens stored in AA regardless of temperature. When compared to the index time point the alpha diversity, as measured by the Shannon index, was decreased on days 2 and 4 in samples stored at RT without AA (Fig. S2). While the mechanism by which AA preserves specimens is unclear, it is certainly useful for reducing diversity changes at high storage temperatures.

We previously characterized differences in the microbiomes in a group of individuals using weighted UniFrac distances as our standard for identifying microbiome differences^35^. In this cohort, we found that the weighted UniFrac distances and Bray Curtis dissimilarity increased through time at RT, but these results were not statistically significant (Fig. 4). However, we did find for samples stored at RT, the weighted UniFrac distances and Bray Curtis dissimilarity in specimens preserved with AA were significantly lower than those stored without. This supports previous data that suggests AA can help preserve the urinary microbiome at RT.

Limitations to this study include the small sample size, which may limit the power to detect statistically significant differences in diversity and taxonomic profiles among the samples. Additionally, we only characterized the urinary microbiomes of asymptomatic women, thus the findings in this study may not be broadly applicable to women with lower urinary tract conditions such as UTIs and incontinence that are known to be associated with altered microbiomes^6,9,36^. The medical histories of the participants were not available in this study; therefore we were unable to assess the potential effect of chronic illnesses such as diabetes and obesity on the urinary microbiome. We also only analyzed voided urine, which has the potential for contributions from vaginal and urethral microbiota. The urine specimens were also collected at varying times during the day, which could have led to differences based on levels of participant food/fluid consumption and/or physical activity. Finally, although we analyzed the AA for contamination, there is always potential for contamination at other times of sample collection and processing that could impact microbiome results. This study did not assess the utility of specimens that have been preserved for extended time periods, such as repositories of frozen specimens, nor did we assess the use of other preservatives. We focused primarily on the AA preservative due to its widespread use in urine microbiome studies^1,6,22,28,37^. Standardization at each of the levels of collection, processing, storage, and analysis is critical to future work.

## Conclusions

Addition of preservative (AA), shorter storage times and colder temperature are generally more favorable conditions for urine samples used in urinary microbiome studies. While it has been general practice of some groups to limit storage times to less than 4 hours on ice or with refrigeration (4 °C) before the addition of AA prior to freezing at −80 °C^1,6,8,28^, this is the first attempt to benchmark preservation and storage methods for urinary microbiome studies. The AA preservative combined with storage temperatures of no higher than 4 °C for periods up to 4 days appears to be optimal for urine microbiome analysis to ensure reproducibility. When neither AA nor temporary refrigeration is available, transfer to −80 °C should occur within 3 hours of collection. These results are encouraging in that urine specimens stored at colder temperatures for shorter time periods prior to storage at −80 °C seem reliable for urine microbiome analysis. Research teams should avoid RT storage without AA because the microbiome is altered rapidly under these conditions. Benchmarking studies such as the one presented here can help to provide a template for storage and preservation conditions to allow greater reproducibility in microbiome studies. Given these findings, we recommend use of AA whenever samples cannot be immediately cooled prior to transfer to −80 °C storage. Additionally, shipping room temperature urine specimens without the addition of AA to a central study site may affect study reproducibility. Samples preserved with AA at RT may be valid for analysis up to 4 days from collection. This has implications for studies considering home collection and shipment to a central facility.

## Methods

### Human participants

We recruited via word-of-mouth and enrolled 10 healthy female volunteers and collected urine from each (Table S1). We obtained approval (Project #170077AW) from the University of California, San Diego administrative panel on human subjects research to conduct the study, and each participant signed an informed consent form indicating their willingness to participate in the study. All methods in this study were performed in accordance with the relevant guidelines and regulations. For 10 of the participants, we collected midstream, voided urine samples. Those specimens were immediately split into 36 separate 200 µl aliquots, with half the aliquots preserved with 20 µl of AA, and the other half stored without preservative (Fig. 1). Each aliquot then was stored at RT, 4 °C or −20 °C for either 1 hour, 3 hours, overnight (approximately 16 hours), 2 days, or 4 days. Specimens then were stored at −80 °C prior to processing for microbiome analysis.

### Processing of 16S rRNA gene amplicons

DNA was extracted from vortexed liquid urine specimens using the Qiagen DNeasy Powersoil kit (Qiagen) and further concentrated using the Zymo gDNA Clean and Concentrate kit (Zymo). Purified and concentrated DNA was subjected to PCR using Kapa Hifi Hotstart Readymix (Kapa Biosystems) with PCR forward primer 5′-TCG TCG GCA GCG TCA GAT GTG TAT AAG AGA CAG CCT ACG GGN GGC WGC AG-3′ and reverse primer 5′-GTC TCG TGG GCT CGG AGA TGT GTA TAA GAG ACA GGA CTA CHV GGG TAT CTA ATC C-3′ to amplify the V3-V4 hypervariable segment of the 16S rRNA gene^27^. We used the following cycling parameters: 95 °C for 3 minutes, followed by 35 cycles of 95 °C for 30 seconds, 55 °C for 30 seconds, 72 °C for 30 seconds, and a final elongation step of 72 °C for 5 minutes. Amplicons were purified with Ampure XP beads (Beckman-Coulter) and visualized using a High Sensitivity DNA Kit on a Bioanalyzer (Agilent Technologies). Molar equivalents were determined for each sample by quantifying the amplicons using a dsDNA High Sensitivity Kit on a Qubit Fluorometer (Thermo Fisher). Samples then were pooled into equal molar proportions and sequenced on the Illumina MiSeq platform (Illumina) yielding 300 bp paired-end reads.

### Analysis of the 16S rRNA gene

We sequenced 13,666,338 high quality reads with an average of 34,166 reads per sample. Sequence reads were analyzed using Quantitative Insights Into Microbial Ecology 2 (QIIME2; available at https://qiime2.org)^38^. Sequences were filtered based on quality to remove phiX reads, chimeric sequences, and were also denoised using the DADA2 pipeline^39^. Samples with less than 10,000 sequences were removed from downstream analyses. Alpha diversity was determined via the number of Observed OTUs and Shannon index generated by QIIME2 core-metrics-results pipeline. The change in alpha diversity for each participant was determined by taking the absolute value of the difference in diversity between each time point and the index time point for each condition. Beta diversity was determined using weighted and unweighted UniFrac distances and Bray Curtis dissimilarity^40,41^. Principal Coordinates analysis plots were generated for weighted and unweighted UniFrac distances using the core-metrics-results pipeline and qiime2R package (available at https://github.com/jbisanz/qiime2R). A PERMANOVA test (999 permutations) was applied to the unweighted UniFrac distances to determine significance between samples treated with and without AA. Taxonomic assignments were performed using the Naïve Bayes classifier based on the Greengenes database^31^. Statistically significant differences in beta diversity metrics, alpha diversity, change in alpha diversity, and the relative abundances of bacterial phyla were determined by Tukey’s Honest Significant Difference test and two-tailed t-tests. Differential abundance testing was performed with the gneiss pipeline^42^.

We characterized individual-specific features of urinary microbiomes of the participants and determined the beta diversity amongst the microbiomes of each participant and time point and visualized the output using Principal Coordinates Analysis. We calculated and compared the Bray Curtis dissimilarity and weighted UniFrac distances between each time point (1 hour, 3 hours, overnight, 2 days, and 4 days) with the index time point (immediately frozen at −80 °C) to evaluate microbiome preservation. We next used differential abundance testing^33^ to identify which time and temperature conditions have the greatest variability in the representation of some microbes with and without AA. We also characterized the change in alpha diversity across time in the urine specimens processed with and without AA, and compared the alpha diversity at each time point to the index time point.

## Supplementary information

Supplementary Figures and Tables

## Data Availability

All sequences are available for download in the Sequence Read Archive under Accession Number PRJNA521072 (https://www.ncbi.nlm.nih.gov/sra/?term=PRJNA521072).
