# Supplementary material for: Benchmarking urine storage and collection conditions for evaluating the female urinary microbiome
Source: Sci Rep. 2019 Sep 16;9:13409. doi: 10.1038/s41598-019-49823-5 (PMC6746804; doi:10.1038/s41598-019-49823-5)
Supplement: Supplementary file 1 — Supplementary Figures and Tables [file 41598_2019_49823_MOESM1_ESM.pdf]

Benchmarking urine storage and collection conditions for evaluating the female urinary microbiome

Carrie E. Jung[1], Jessica Chopyk[2], Ji Hyun Shin[2], Emily S. Lukacz[1], Linda Brubaker[1], Leila K. Schwanemann[2], Rob Knight[3], Alan J. Wolfe[4], and David T. Pride[2,5*]

Supplementary Figures and Tables

Figure S1

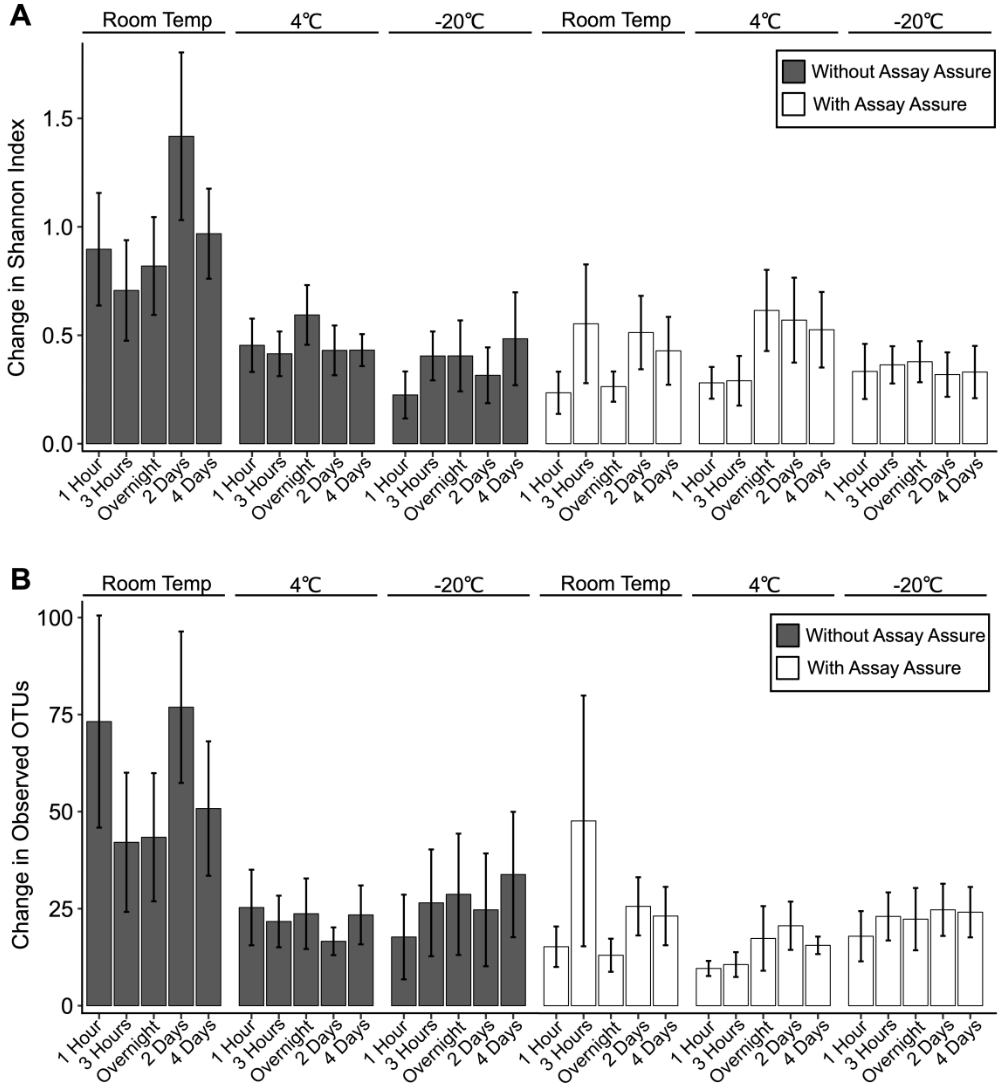

Figure S1: Bar graphs representing mean changes in the (A) Shannon Index (±standard error) and (B) Observed OTUs values (±standard error) compared to the index time point (time point where urine specimens were collected and then immediately frozen at -80°C) for urine specimens from all participants. Urine specimens preserved in AA are demonstrated by white bars and specimens without preservative are shown by gray bars. The mean change mean changes in the Shannon Index and Observed OTUs values are shown on the y-axis and the x-axis represents the time specimens were stored prior to transfer to -80°C.

Figure S2

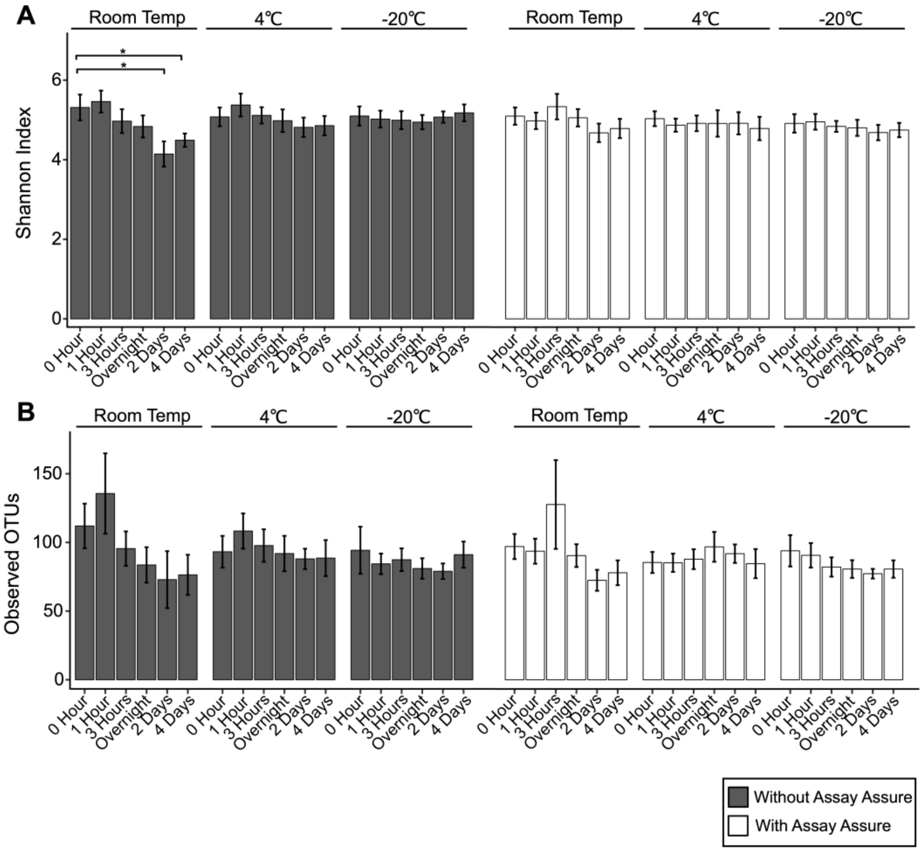

Figure S2: Bar graphs of the (A) Shannon index (±standard error) and (B) Observed OTUs (±standard error) calculated for specimens stored at RT, 4°C, and -20°C with and without AA. Urine specimens preserved in AA are demonstrated by the white bars and specimens without preservative are shown by gray bars. The Shannon index and number of Observed OTUs are shown on the y-axis and the x-axis represents the time specimens were stored prior to transfer to -80°C. The '*' represents statistically significant values (p-values ≤0.05) using a two-tailed t-test.

Figure S3

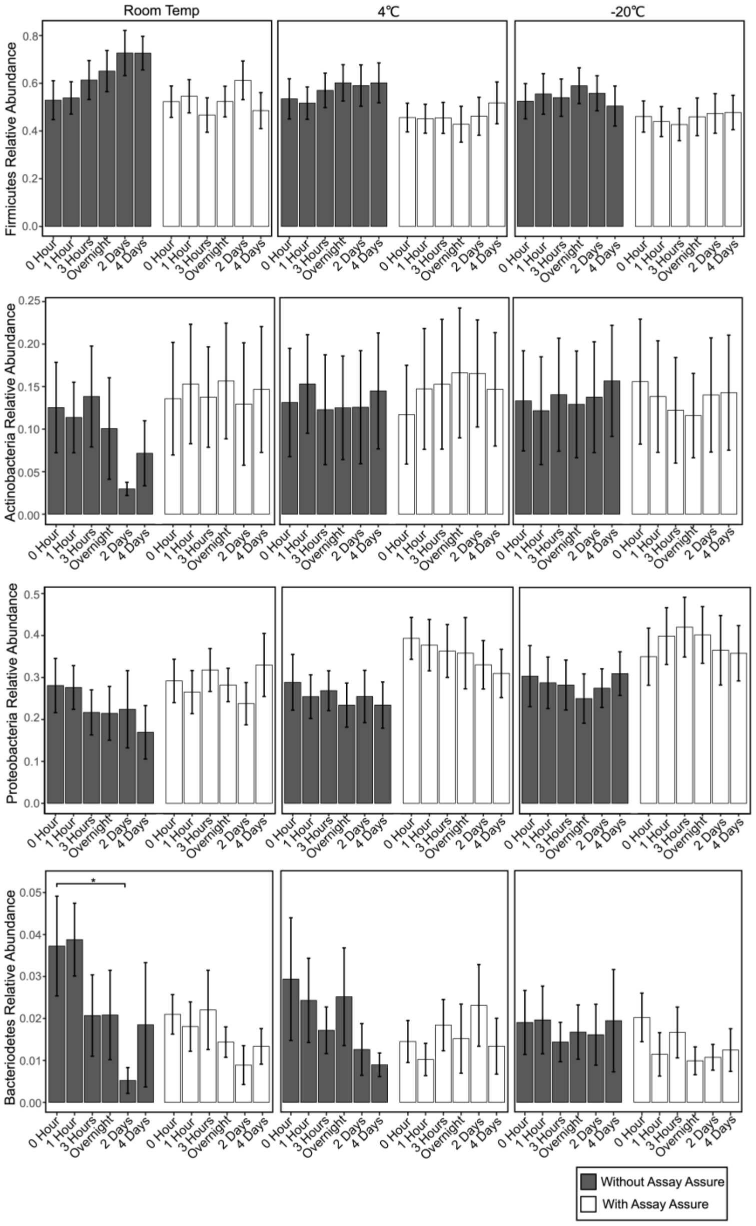

Figure S3: Bar graphs of the relative abundance (±standard error) of the dominant bacterial phyla for specimens stored at RT, 4°C, and -20°C with and without AA. Urine specimens preserved in AA are demonstrated by the white bars and specimens without preservative are shown by gray bars. The relative abundance of *Firmicutes*, *Actinobacteria*, *Proteobacteria*, and *Bacteriodetes* are shown on the y-axis and the x-axis represents the time specimens were stored prior to transfer to -80°C. The '*' represents statistically significant values (p-values ≤0.05) using a two-tailed t-test.

Figure S4

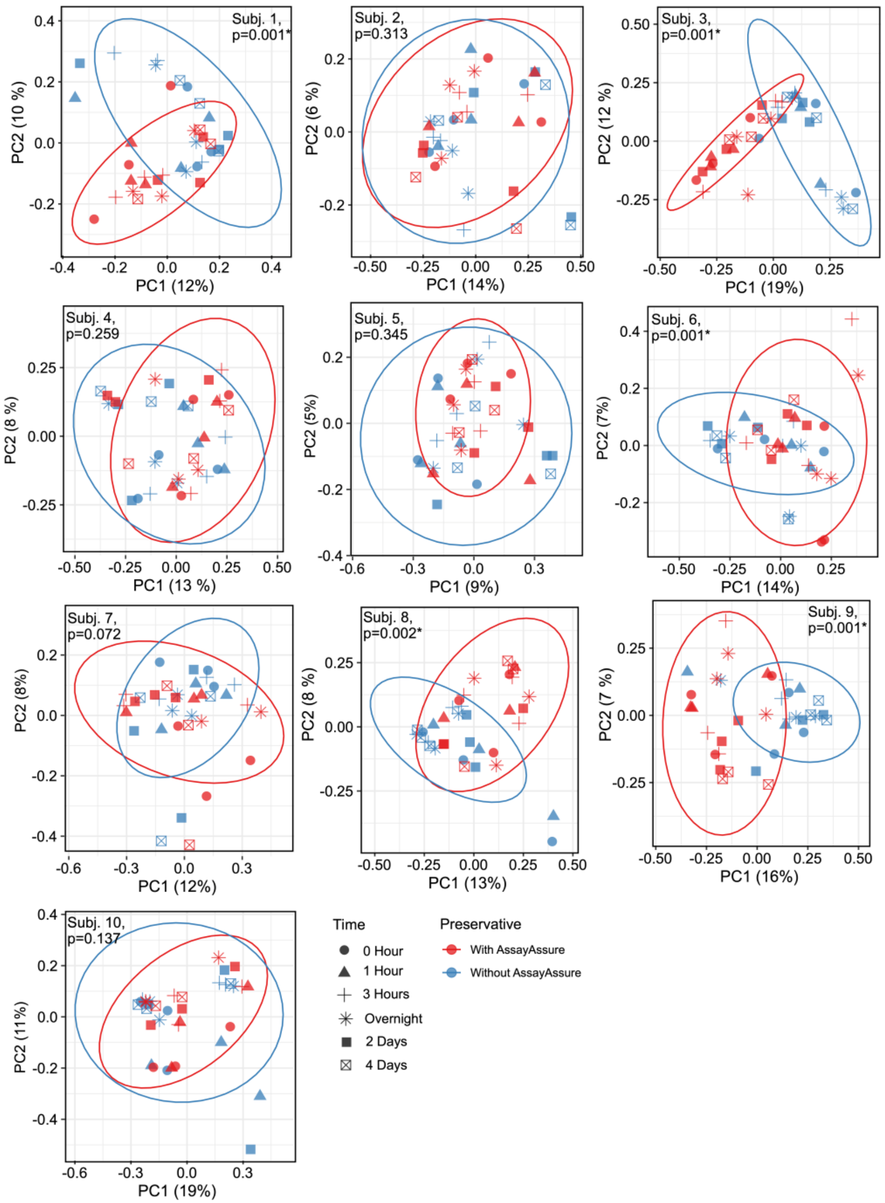

Figure S4: Principal coordinates analysis based on unweighted UniFrac distances of urine specimens for each of the 10 participants studied. Urine specimens preserved in AA are demonstrated by the red points and specimens without preservative are shown by blue points. Ellipses are drawn at 95% confidence intervals for specimens stored with and without AA. Shape of the point reflects the time specimens were stored prior to transfer to -80°C. Statistical differences were determined using a PERMANOVA with 999 permutations.

Figure S5

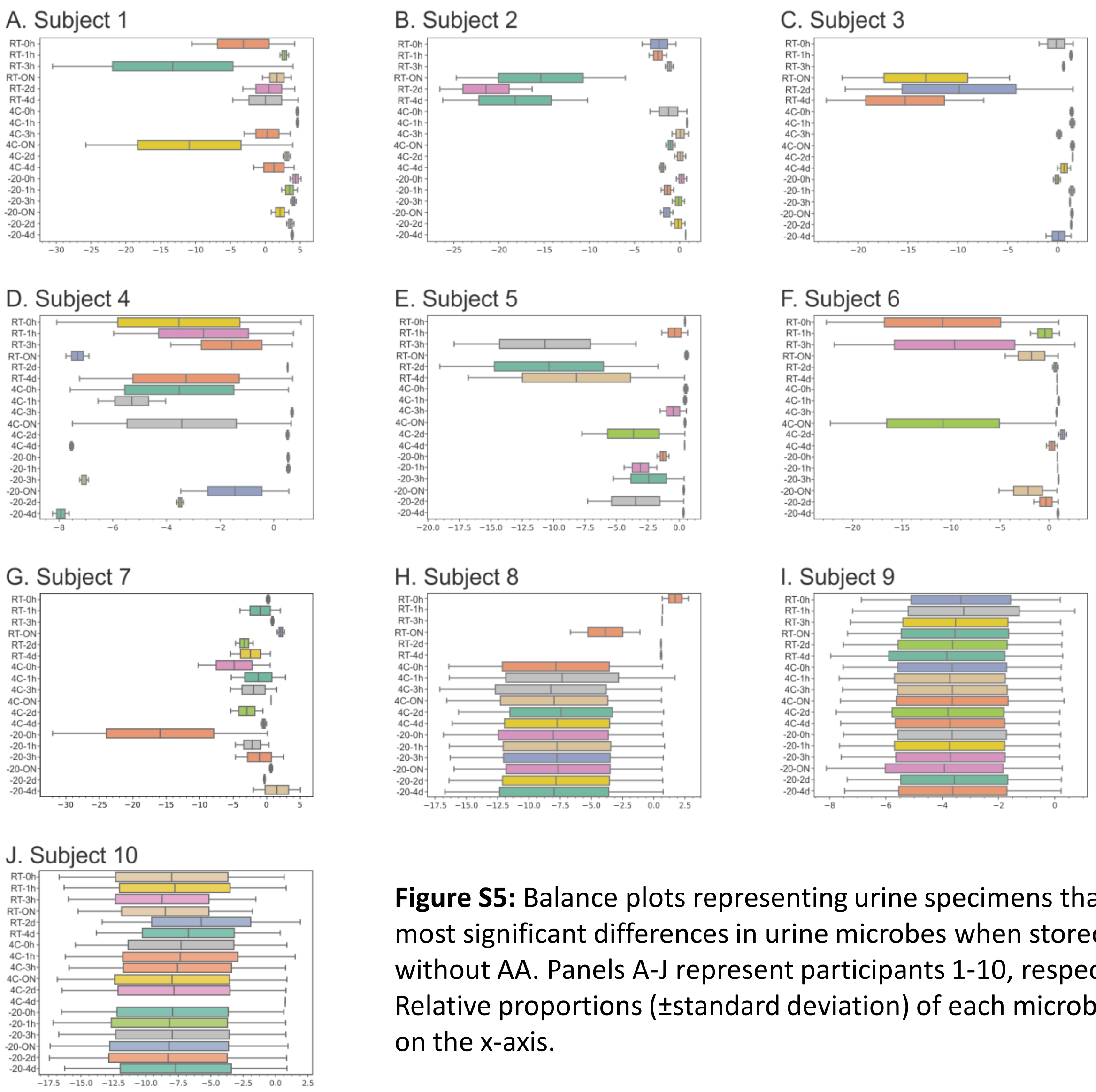

Figure S5: Balance plots representing urine specimens that have the most significant differences in urine microbes when stored with and without AA. Panels A-J represent participants 1-10, respectively. Relative proportions (±standard deviation) of each microbe are shown on the x-axis.

Table S1. Human Participant Demographics

Participant	Age	Menopausal Status	Race
1	31	Pre	Caucasian/Asian
2	55	Post	Caucasian
3	38	Pre	Latino/Asian
4	32	Pre	Asian
5	62	Post	Caucasian
6	47	Pre	Caucasian
7	23	Pre	Asian
8	33	Pre	Caucasian
9	33	Pre	Caucasian
10	50	Post	Asian

Table S2: Differentially abundant taxa in participants

Taxon[a]	Number of participants	Subject #[b,c]
Bacillaceae	7	↑1, ↑3, ↑4, ↓7, ↓8, ↓9, ↓10
Stenotrophomonas	6	↑1, ↑ 2,↑ 5,↑ 6,↑ 7,↓ 9
Delftia	6	↑1, ↑2, ↑4, ↑8, ↑↓9, ↑↓10
Lactobacillus	4	↑3, ↑4, ↑5, ↑6
Gardnerella	4	↑3, ↓4, ↑5, ↑6
Anaerobacillus	4	↑4, ↓8, ↓9, ↓10
Enterococcus	3	↑2, ↓3, ↓5
Klebsiella	3	↓7, ↓8, ↓10
Brucellaceae	3	↑6, ↑7, ↓9
Escherichia	2	↓1, ↓9
Streptococcus	2	↑4, ↓10
Propionibacterium	2	↑5, ↓8
Ureaplasma	2	↑1, ↑7
Dialister	2	↑3, ↑4
Microbacterium	2	↑2, ↑7
Rhizobiaceae	2	↑7, ↓9
Rhodococcus	2	↑7, ↓10
Bacillus	2	↓6, ↓9
Prevotella	2	↑3, ↓7
Sphingomonadales	1	↑1
Bradyrhizobia	1	↑1
Faecalibacterium	1	↓1
Roseburia	1	↓1
Ruminococcus	1	↓1
Akkermansia	1	↓1
Bacteroidales	1	↓1
Bacteroides	1	↓1
Corynebacterium	1	↑2
Ralstonia	1	↑2
Facklamia	1	↑2
Lactococcus	1	↓2
Streptococcaceae	1	↓3
Pseudomonas	1	↑4
Meiothermus	1	↑4
Neisseriaceae	1	↑4
Kaistobacter	1	↑5
Burkholderia	1	↑5
Bacilli	1	↑6
Citrobacter	1	↑6
Streptophyta	1	↑6

Veillonellaceae	1	↓6
Thermicanus	1	↓6
Cloacibacterium	1	↓6
Hydrogenophilus	1	↓6
Porphyromonadaceae	1	↓6
Sphingomonas	1	↑7
Agrobacterium	1	↑7
Actinomycetales	1	↓7
Variovorax	1	↓7
Finegoldia	1	↓7

[d]Differentially abundant taxa with and without AA
[b]Up arrows indicate greater abundance in corresponding taxa with AA
[c]Up and down arrows together indicate taxa with representative OTUs that increased and representative OTUs that decreased in abundance with AA
